# Genomic Epidemiology of *Streptococcus suis* Sequence Type 7 Sporadic Infections in the Guangxi Zhuang Autonomous Region of China

**DOI:** 10.3390/pathogens8040187

**Published:** 2019-10-12

**Authors:** Mingliu Wang, Pengcheng Du, Jianping Wang, Ruiting Lan, Jun Huang, Ming Luo, Yan Jiang, Jun Zeng, Yi Quan, Zhaohui Shi, Han Zheng

**Affiliations:** 1 Guangxi Zhuang Autonomous Region Center for Disease Prevention and Control, Nanning 530028, China; wmlml@163.com (M.W.); jun007210@163.com (J.H.); zengjunhf@126.com (J.Z.); cdcquanyi@126.com (Y.Q.); 2 Beijing Key Laboratory of Emerging Infectious Diseases, Institute of Infectious Diseases, Beijing Ditan Hospital, Capital Medical University, Beijing 100015, China; dupengcheng126@126.com; 3 State Key Laboratory of Infectious Disease Prevention and Control, National Institute for Communicable Disease Control and Prevention, Chinese Center for Disease Control and Prevention, Changping, Beijing 102206, China; wangjianping@icdc.cn; 4 School of Biotechnology and Biomolecular Sciences, University of New South Wales, Sydney, NSW 2052, Australia; r.lan@unsw.edu.au; 5 Yulin Center for Disease Prevention and Control, Yulin 537000, China; lm13877560799@163.com; 6 Qinzhou Center for Disease Prevention and Control, Qinzhou 535099, China; 15107876699@139.com; 7 Guilin Center for Disease Prevention and Control, Guilin 541001, China; shizhaohui.66@163.com

**Keywords:** *Streptococcus suis*, sequence type 7, sporadic strain, serotype 14, phylogenetic structure, comparative genomes

## Abstract

*Streptococcus suis* is an important zoonotic pathogen. Serotype 2 and sequence type (ST) 1 are the most frequently reported strains in both infected humans and pigs. ST7 is only endemic to China, and it was responsible for outbreaks in 1998 and 2005 in China. In the present study, 38 sporadic ST7 *S. suis* strains, which mostly caused sepsis, were collected from patients in the Guangxi Zhuang Autonomous Region (GX) between 2007 and 2018. Of 38 sporadic ST7 strains, serotype 14 was the most frequent (27 strains, 71.1%), followed by serotype 2 (11 strains, 28.9%). The phylogenetic structure of the ST7 population, including epidemic and sporadic ST7 strains, was constructed using mutational single-nucleotide polymorphisms (SNPs). High diversity within the ST7 population was revealed and divided into five lineages. Only one sporadic ST7 strain, GX14, from a *Streptococcal* toxic-shock-like syndrome (STSLS) patient was clustered into the same lineage as the epidemic strains. GX14 and the epidemic strains diverged in 1974. The sporadic ST7 strains of GX were mainly clustered into lineage 5, which emerged in 1980. Comparing to genome of epidemic strain, the major differences in genome of sporadic ST7 strains of GX was the absence of 89 kb pathogenicity island (PAI) specific to epidemic strain and insertion of 128 kb ICE_phage tandem MGE or ICE portion of the MGE. These mobile elements play a significant role in the horizontal transfer of antibiotic resistance genes in sporadic ST7 strains. Our results enhanced the understanding of the evolution of the ST7 strains and their ability to cause life-threatening infections in humans.

## 1. Introduction

*Streptococcus suis* is an important zoonotic pathogen, with pigs as the main reservoir. Patients get infected through close contact with infected pigs or pork-derived products. The majority of *S. suis* infections are reported in Thailand, Vietnam, and China [1]. Moreover, *S. suis* has been identified as the third leading cause of bacterial meningitis in adults in Vietnam and Thailand [1,2,3,4]. Acute meningitis is the most common clinical feature of sporadic infections. Distinctively, most cases in China were from outbreaks [2]. A large outbreak in the summer of 2005 in Sichuan Province resulted in 215 cases and 39 deaths [5,6], whereas a smaller outbreak, previously overlooked, occurred in Jiangsu Province in 1998 with 25 cases and 14 deaths [5]. These two outbreaks were characterized by high rates of *Streptococcal* toxic-shock-like syndrome (STSLS), which is rare in sporadic cases. Multilocus sequence typing (MLST) analysis showed that the two outbreaks were caused by sequence type (ST) 7 strains and were referred to as epidemic strains which derived from ST1 by acquiring 5 genomic islands [6,7]. By minimum core genome sequence typing (MCG), epidemic strains were typed into MCG 1 which also contained ST1, ST6, ST11, ST17 and ST81 [8]. The epidemic strains were specifically distinguished from the other STs of MCG1 by the 13 single-nucleotide polymorphisms (SNPs) [8].

High level of genetic heterogeneity was observed among *S. suis* strains within the same ST [9,10]. Defining the phylogenetic relationships among different *S. suis* ST7 strains will contribute to a better understanding of their emergence and evolution as important pathogens in humans. However, the population structure and genetic diversity of the ST7 strains remain poorly understood, given that *S. suis* ST7 strains have been isolated only in China and most strains were derived from outbreaks [11]. In the present study, 38 *S. suis* ST7 strains were collected from patients occurred sporadically between 2007 and 2018 in GX. These strains were referred to as sporadic ST7 strains. We sequenced the genomes of the 38 sporadic ST7 strains of GX and evaluated the phylogenetic relationships among the ST7 epidemic and sporadic strains using genomic data. The clinical manifestations, virulence gene genotypes, antibiotic resistance (AR) genes, and corresponding antimicrobial susceptibility profiles of these sporadic ST7 strains of GX were also determined.

## 2. Results

### 2.1. MCG Analysis and Serotyping of S. suis ST7 Sporadic Strains

By MCG typing, all 38 ST7 strains from GX were typed to MCG 1. Interestingly, the 13 SNPs used to track the epidemic strains could also distinguish all the sporadic ST7 strains from the epidemic strains [8]. By serotyping, the 38 ST7 GX strains were divided into two serotypes with 27 (71.1%) serotype 14 isolates and 11 (28.9%) serotype 2 isolates.

### 2.2. Phylogenetic Relationships among the S. suis ST7 Sporadic and Epidemic Strains

The sequencing depth of the 38 strains was 652 ± 94 in average. SC84 was used as a reference for mapping and 4735 SNPs were identified among them, ranging from 1 to 1423 high quality SNPs per genome.

**Figure 1:**
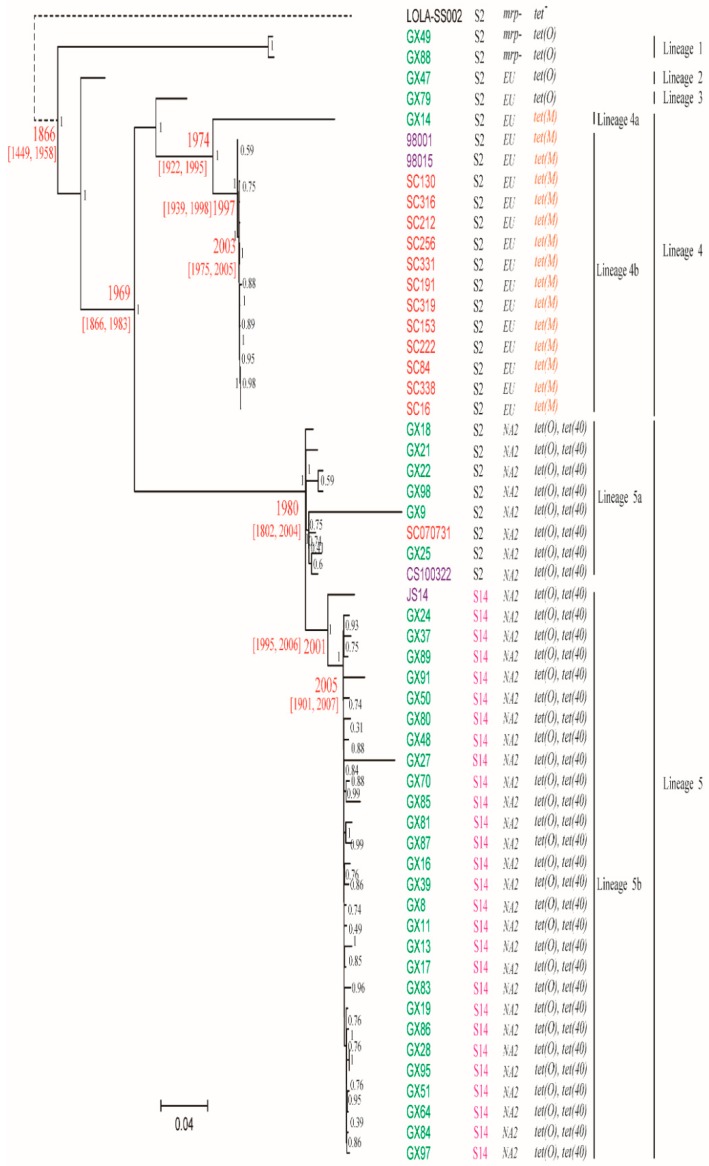
A maximum-likelihood phylogenetic tree of ST7 population strains based on mutational SNPs differences across the whole core genome. The ST1 strain LOLA-SS002 was used as an outgroup to root the tree. The strains are colored on based on the regions of the isolation. Red is Sichuan Province, purple is Jiangsu Province and green is GX. Serotype 14 and *tet*(M) gene were colored in pink and orange red, respectively. The dates shown in red are the median estimates for the indicated nodes and corresponding 95% confidence intervals, taken from the results of the BEAST analysis. The bootstrap values were added in each node in black. The scale is given as the number of substitutions per variable site.

An ST1 strain, LOLA-SS0002, was used as an out-group of the phylogenetic tree. We classified ST7 population into five lineages. A total of 1785 SNPs supported the classification of the lineages (Figure 1).

Lineages 1, 2 and 3 contained 2, 1 and 1 sporadic serotype 2 GX strains, respectively, which appeared to be early diverged lineages (Figure 1). Lineage 4 contained one sporadic serotype 2 strain GX14 and all epidemic serotype 2 strains, with the latter being clustered together and separated from GX14 by 1582 SNPs. The remaining 33 sporadic GX strains and three complete genomes of sporadic ST7 strains were clustered into lineage 5. 

Within the lineage, serotype 2 strains and serotype 14 strains were separated as two sub-lineage 5a and 5b with 70 SNPs separating them. It is clear that serotype 14 strains shared a common origin and were derived from a serotype 2 strain. The serotype 14 strain JS14 from Jiangsu Province appeared to have diverged from serotype 14 strains from GX with 56 SNPs specific to JS14.

We used BEAST to estimate the divergence time of the main lineages. The rate of SNP accumulation in core genome was estimated to be 1.95 SNPs year^−1^ (95% confidence interval [CI] 0.34–3.1), which was similar to 1.8 SNPs year^−1^ in epidemic strains [12]. Based on the accumulation rate of 1.95 SNPs year^−1^, the most recent common ancestor of the epidemic and sporadic ST7 strains dated back to 1866. The serotype 14 ST7 lineage arose in 2001 and likely spread in GX in 2005. Lineage 4 which gave rise to the epidemic lineage (lineage 4b) arose in 1974, while lineage 5 which gave rise to the serotype 14 lineage arose in 1980. The time estimates were consistent with those in our previous study on the divergence of the outbreak strains [12].

### 2.3. Geographic Distribution of S. suis ST7 Sporadic Strains

**Figure 2:**
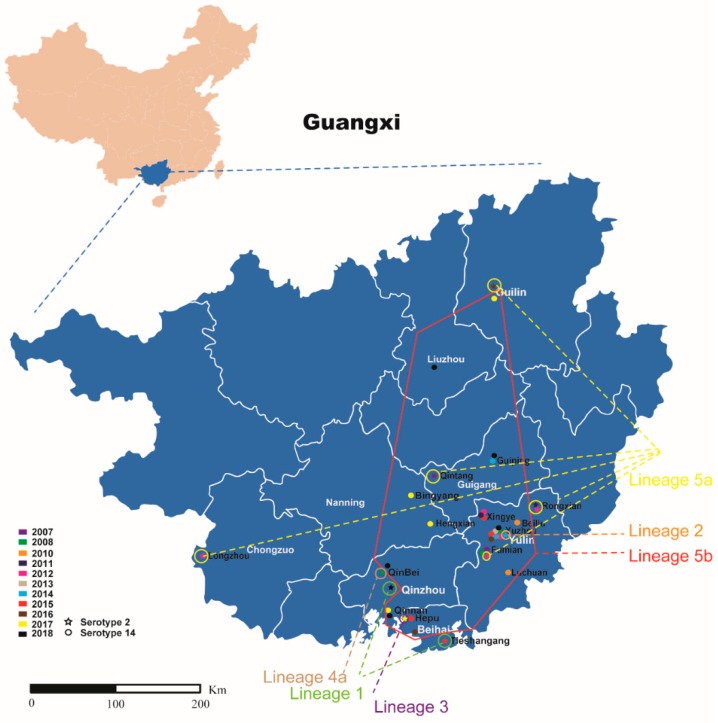
The geographic spread of the sporadic ST7 strains of GX. Strains of different lineages are indicated in different colors (lineage 1, 2, 3, 4a, 5a and 5b is green, orange, purple, brown, yellow and red, respectively). Stars in different colors on the map represent serotype 2 isolated in different years; Cycles in different colors on the map represent serotype 14 isolated in different years.

There was a wide geographic distribution of the strains within the same lineage. Two lineage 1 strains were found in two different cities, namely, Beihai City and Qinzhou City. Six lineage 5a strains were distributed in four cities, and 28 lineage 5b strains were distributed in seven cities (Figure 2).

### 2.4. Virulence Genes of S. suis ST7 Sporadic Strains 

All the ST7 GX strains were positive for the *ef* and *sly* genes. Except for two lineage 1 strains, all were positive for the *mrp* gene. SSU05_0473, *neuB*, *neuC*, SpyM3-0908, *rgg*, *nadR*, *ofs*, *sao* and *revS*, present in highly pathogenic strains [11,13], were present in all sporadic ST7 GX strains. Additionally, the 8 regions of difference (RD) [11], which were previously identified to be preferentially present in highly pathogenic strains were also present in sporadic ST7 GX strains (excluding RD17 coding serotype 2 cps locus, normally absent in serotype 14 strains).

### 2.5. Whole Genome Synteny Analysis 

**Figure 3:**
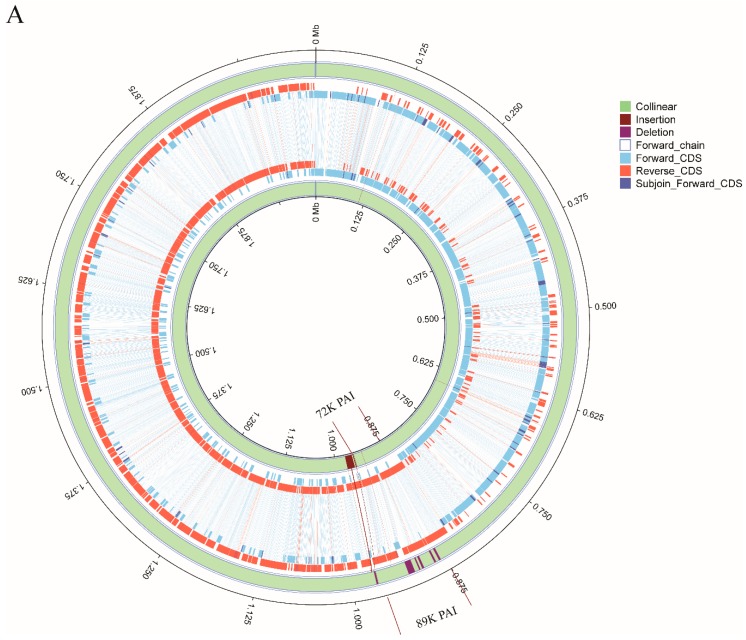

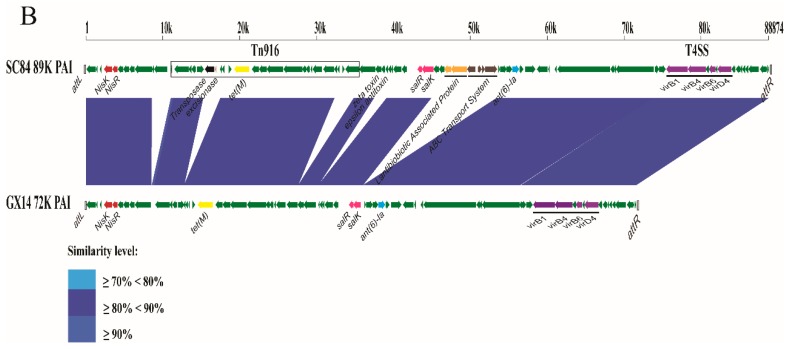
(**A**). Mauve alignment and structure variation of the genome of sporadic ST7 GX14 and epidemic strain SC84. The syntenic regions and unique regions in the genomes are shown as corresponding colored areas. The inner and outer circle is GX14 and SC84, respectively. (**B**). Schematic comparison of 72K PAI in the study and 89K PAI. The direction of the arrow indicates the direction of transcription. Regions of >70% identity were marked by blue shading. The AR genes, 2-component signal-transduction systems, type IV secretion system, lantibiotic biosynthesis cluster and ABC transport system were indicated by different colors. Tn916 was highlighted in a black box. The *att* sites are located in the flanking region of PAIs.

**Figure 4:**
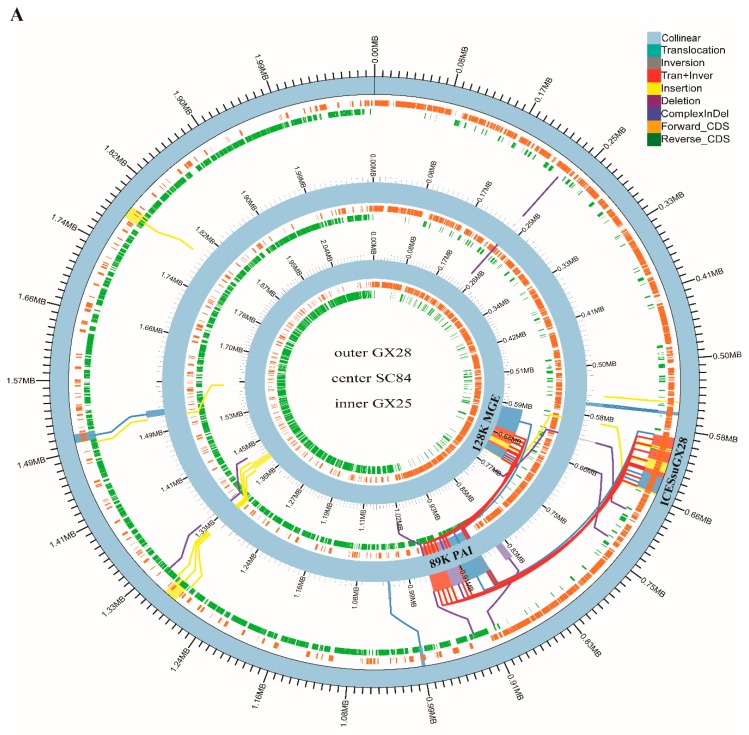

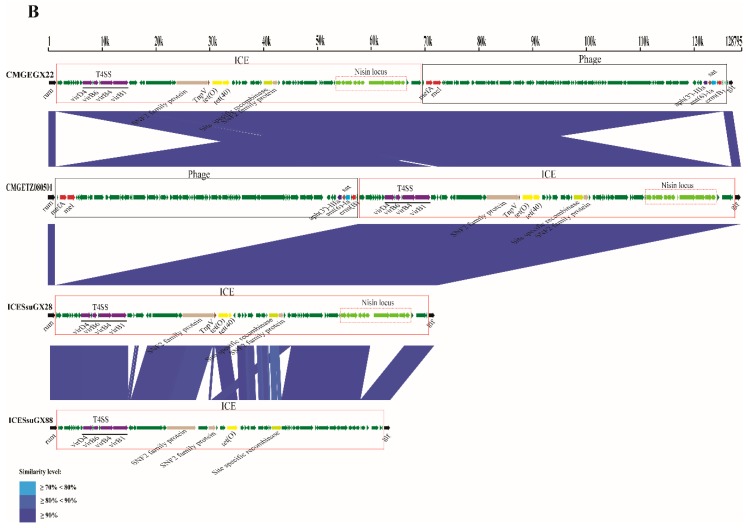
(**A**). Mauve alignment and structure variation of the genome of sporadic ST7 GX25, GX28 and epidemic strain SC84. The syntenic regions and unique regions in the genomes are shown as corresponding colored areas. (**B**). Schematic comparison of ICEs and ICE-phage tandem MGE in the study. The direction of the arrow indicates the direction of transcription. Regions of >70% identity were marked by blue shading. The AR genes were indicated by different colors. ICE and phage were highlighted in a red and black box, respectively.

To gain insights into the genomic differences between sporadic ST7 strains and epidemic strains, complete genomes of lineage 4 strain GX14, lineage 5a strain GX25 and lineage 5b strain GX28 were firstly compared to that of SC84. A high degree of conservation was noted in the genome organizations and sequences between epidemic and two sporadic ST7 strains, major differences among them in that: (i) an 89 kb pathogenicity island (PAI) specific to epidemic strain [14] is absent in Lineage 5 strain GX25 and GX28. Only Lineage 4 strain GX14 harbored a similar island with 99.3% identity and 80% coverage with the 89K PAI at the nucleotide level, which we designated as the 72K PAI (Figure 3A,B). The 72K PAI was inserted into the same site as the 89K PAI between *rplL* and *hdy* and harbored a 15-bp *att* sequence 5′-TTATTTAAGAGTAAC-3′ at both ends of the island. The *NisK*-*NisR*-like and *SalR*-*Sal*K-like 2-component signal transduction systems of the 89K PAI, which may contribute to the virulence of the epidemic strain, were also present in the 72K PAI. An intact type IV secretion system (T4SS) containing the *VirB*1, *VirB*4, *VirB*6, and *VirD*4 genes identified in the 72K PAI was identical to that of the 89K PAI. *tet(M)* and *ant6ia* genes were present in both PAIs. The distinct differences between the two PAIs revealed that a large lantibiotic biosynthesis cluster and an ABC transport system carried by the 89K PAI were absent in the 72K PAI. 

(ii) a 128 kb ICE_phage tandem MGE identical to CMGETZ080501 [15] exists in GX25 but is absent in the epidemic strain SC84 (Figure 4A). Multiple tandem AR genes were present in these MGEs, including *tet(O)- tet(40)*, *mefA* - *mel* and *aph(3’)-IIIa-sat4-erm(B)-ant6ia* (Figure 4B). The *tet(O)*and *tet(40)* genes encoded for tetracyclines resistance. The *ermb*, *mef(A)* and *mel* genes were responsible for the resistance to macrolides and lincosamide. The aminoglycoside O-nucleotidylyltransferase *ant6ia* gene conferred resistance to streptomycin, while the aminoglycoside O-phosphotransferase *aph(3’)-IIIa* gene primarily inactivated kanamycin. The *sat4* gene conferred resistance to streptothricin. In addition, only ICE portion of CMGETZ080501 was present in GX28, named ICESsuGX28 (Figure 4A). ICESsuGX28 harbored tandem *tet(O)- tet(40)* genes (Figure 4B). Both of elements were inserted into same site between *rum* and *glf* genes. All of them harbored a 14 bp *att* sequence 5′-CACGTGGAGTGCGT-3′ and 5′-CACATAGAAGTTGT-3′ in 5′ and 3′ side of region, respectively.

### 2.6. Identification of Lineage-related Genetic Characteristics 

In the study, GX strains from same lineage possessed similar genetic elements and features:

(i) 128 kb MGE harbored in lineage 5a strains GX25 were also present in lineage 5a strains GX9, GX21, GX24 and GX87. Interestingly, both GX22 and GX98 harbored similar 128K MGE, which was named CMGEGX22. Compared to that of CMGETZ080501, the arrangement of the ICE and phage portions was reversed in the CMGEGX22 (Table 1, Figure 4B). 

(ii) ICESsuGX28 were present in all GX strains of lineage 5b (Table 1). Different from GX strains of lineage 5b, three GX strains of lineage 1 and 2 harbored a similar ICE to ICESsuGX28, named ICESsuGX88 (Table 1, Figure 4B). The *tet*(40) gene was absent in ICESsuGX88. A nonsense mutation, an insertion of an A base at position 390, was found in the *tet*(40) gene of all the GX strains in lineage 5b. 

(iii) Except for two lineage 1 GX strains, all were positive for the *mrp* gene. Based on the sequence variation in the central portion of the *mrp* gene, three subtypes, namely, EU, NA1, and NA2 have been reported [16]. All the GX strains of lineages 2, 3, and 4 harbored the EU subtype, whereas all GX strains of lineage 5 harbored the NA2 subtype (Figure 1).

(iv) All strains from STSLS patients were clustered into lineage 4. All 28 lineage 4 specific genes were located in 89K and 72K PAIs 

### 2.7. Antimicrobial Susceptibility Profiles

In addition to tandem AR genes in tandem MGEs and ICE, multiple tandem AR genes were also present in the chromosome of 10 GX strains from lineage 5b, including *erm(B)-ant6ia*- *spw* (GX28, GX51, GX64, GX84, GX86, GX91, GX95 and GX97), *ant6ia* - *aac(6′)Ie-aph(2″)Ia* - *dfr*G (GX39) and *ant6ia*- *aac(6′)Ie-aph(2″)Ia* - *erm(B)* (GX87). In additional, *aph(3″)IIIa -ant6ia*- *sat4* was present in the chromosome of GX79 from lineage 3.

Based on the detection of the AR genes, we tested all the strains for susceptibility to penicillin G, cefaclor, tetracycline, erythromycin, azithromycin, clindamycin, streptomycin, kanamycin, spectinomycin, gentamicin, and trimethoprim–sulfamethoxazole. All the strains were susceptible to penicillin G and cefaclor. By contrast, all the strains were resistant to tetracycline with MICs between 16 and 32 μg/mL. Concomitant resistance to erythromycin and clindamycin was observed in strains carrying *erm*(B) as reported in previous studies [17,18]. The MICs of both antibiotics were >256 μg/mL.

High degrees of kanamycin (MIC >256 μg/mL), spectinomycin (MIC >1024 μg/mL), and gentamicin (MIC >256 μg/mL) resistance were found in strains carrying *aph3-iiia*, *spw*, and *aac(6′)Ie-aph(2″)Ia* genes, consistent with previous studies [19,20,21], respectively. The MIC of streptomycin was >1024 μg/mL in 17 strains carrying the *ant6ia* gene. GX39 carrying the *dfrG* gene was resistant to trimethoprim–sulfamethoxazole with an MIC >32 μg/mL.

Two strains, GX81 and GX87, were resistant to erythromycin, azithromycin and clindamycin with MICs of 48 μg/mL, >256 μg/mL, and >256 μg/mL, respectively, but they did not harbor known AR genes. Similarly, strain GX9 had an MIC of >32 μg/mL against trimethoprim–sulfamethoxazole. It also did not harbor known AR genes (Table 1).

## 3. Discussion

MLST has been wildly used to genetically classify *S. suis* strains. To date, ST1 and ST7 have been predominately reported to be responsible for human infections in China. Different from ST1 as most common culprit of the human cases worldwide, the ST7 strains were endemic to China and responsible for two deadly outbreaks in 1998 and 2005 [6]. However, few sporadic cases caused by ST7 strains have been studied, even though ST7 strains have been usually isolated from diseased pigs in China [22,23]. In China, sporadic ST7 strains from patients were mainly reported in GX and Guangdong province [24]. In this study, 38 sporadic cases infected with ST7 strains were collected in GX between 2007 and 2018. Our study revealed that the majority of the sporadic cases were caused by serotype 14, derived from serotype 2 within the ST7 strain. Worldwide, serotype 2 is the most frequently reported serotype at 74.7%, followed by serotype 14 at only 2.0% [2]. Among the 177 *S. suis* strains isolated from patients in Thailand, 165 (93.2%) and 12 (6.8%) were identified as serotype 2 and 14, respectively [25]. 

Our phylogenetic analysis of the epidemic and sporadic ST7 strains revealed a high level of genomic heterogeneity among the strains, which were divided into five lineages. All the sporadic ST7 strains could be clearly separated from the epidemic strains by 13 SNPs specific to the epidemic strains as found in our previous study [8]. 

Sporadic GX strains were mainly clustered into lineage 5 which contained two serotypes. Interestingly, three sporadic strains from Jiangsu and Sichuan Provinces were also clustered with GX sporadic strains. Our data suggested that lineage 5 emerged in 1980, and then spread across China. The epidemic strains were previously found to have likely spread from Jiangsu to Sichuan Province via an inter-provincial spread [12]. It is likely that the trans-provincial transportation of breeder pigs played a key role in the spread of ST7 strains across China.

Serotype 2 and 14 strains in lineage 5 were clustered into separate sub-lineages. In particular serotype 14 strains were closely related and belonged to the same sub-lineage. It is most likely that the horizontal transfer of *cps* genes resulted in the replacement of the serotype 2 *cps* gene cluster by the serotype 14 *cps* gene cluster [26,27]. Since all serotype 14 strains shared a common origin and were very closely related, the replacement occurred only once and was estimated to have occurred in 2005. The exchange of serotypes has further diversified the ST7 population.

All epidemic strains were clustered into lineage 4b. The ancestor of lineage 4 strains was estimated to have emerged in 1974. In our previous study, the estimated time of the most recent common ancestor for the epidemic strains in China was May 1996 [12], indicating that it took over 20 years for ST7 to evolve into an epidemic strain capable of causing severe outbreaks. One sporadic ST7 strain, GX14, was clustered in the same lineage as the epidemic strains that caused STSLS, which was characteristic of the epidemic cases. GX14 differs from the epidemic strains by the presence of a 72K PAI, whereas the epidemic strains harbor an 89K PAI [14]. GX14 appears to be ancestral to the epidemic strains. The 89K PAI may contribute to the development of STSLS by the T4SS-like system and two-component signal transduction systems (TCSTS) [28,29,30]. Both PAIs contained an identical T4SS-like system and two-component signal transduction system but differ in that a large lantibiotic biosynthesis cluster and ABC transporter system carried by the 89K PAI. However, the cluster cannot produce a functional antibiotic due to mutation [31]. It is likely that the most recent common ancestor of GX14 and the epidemic strains (lineage 4) acquired the 72K PAI, and the epidemic lineage further acquired other genes to become the 89K PAI. There are at least five insertions in the 89K PAI compared to the 72 K PAI, suggesting the recent multiple acquisitions of genes. All 28 lineage 4 specific genes were located in 89K and 72K PAIs. These findings further emphasize the fact that these PAIs have contributed to their increased virulence and the capacity of causing more severe outbreaks.

The most prevalent genotype of the *mrp* gene in the sporadic ST7 GX strains was NA2, whereas the genotype of the epidemic strains was EU. The difference in the *mrp* genotype has further confirmed the different origins of the epidemic and major sporadic ST7 strains. It is noteworthy that the NA2 subtype was also common in the ST7 strains from diseased pigs [22] and non-ST7 strains of MCG2 [32] in China.

The differences in genome organizations between epidemic and sporadic ST7 strains were caused by acquiring and deletion of MGEs. MGEs also play a significant role in the horizontal transfer of AR genes in *Streptococcus* [15]. ICE_phage tandem MGE carrying multiple tandem AR genes were found in GX strains from lineage 5a. The ICE portion carried *tet*(O) in tandem with *tet*(40), whereas the phage portion carried an *erm*(B)-containing MAS-like fragment and the *mel*/*mef(A)* cassette. They conferred the tetracycline–macrolide–lincosamide–aminoglycoside antibiotics resistance to host strains. The phage portion was absent in remaining GX strains. Our data indicated that the integration of the phage enhanced MGE diversity and played a key role in the dissemination of AR genes in the sporadic ST7 strains. 

Different from the sporadic ST7 strains of lineage 1, 2, 3 and 5, only the lineage 4 strains carried the tetracycline resistance gene *tet*(M) by transposon *Tn916* (Figure 3). It is noteworthy that *tet*(M) is a prevalent tetracycline resistance gene in *S. suis* strains from sporadic meningitis patients in Vietnam, which is also associated with the presence of *Tn916*-like elements [33].

Two sporadic ST7 GX strains were resistant to trimethoprim–sulfamethoxazole. To our knowledge, this is the first case of *S. suis* strains from patients that were resistant to trimethoprim–sulfamethoxazole. The *dfrG* gene, which confers resistance to trimethoprim [34], contributed to the resistant phenotype of GX39. No known trimethoprim–sulfamethoxazole resistant determinants were identified in GX9. A similar phenomenon was also found in GX81 and GX88 of lineage 5b. Despite the lack of known macrolide and lincosamide resistant determinants, they were resistant to erythromycin, azithromycin and clindamycin. Moreover, mutations in the genes coding for L4 and L22 ribosomal proteins and for 23S rRNA were not identified in their genomes. Novel macrolide, lincosamide and sulfonamide resistance determinants may be present in the three strains, and further studies are needed to address this. 

In conclusion, sporadic ST7 *S suis* infections in GX were predominantly due to serotype 14 strains, in contrast to outbreaks in China, which were caused by serotype 2 strains. The major symptoms also differed, with sepsis for sporadic cases. The ST7 serotype 14 was derived from a ST7 serotype 2 strain about 18 years ago (2001). The sporadic strains could be divided into five lineages. Only one sporadic GX strain fell into lineage 4, and it had the potential to cause an outbreak. These results have enhanced our understanding of the evolution of the ST7 strains and their ability to cause life-threatening infections in humans. In addition, multiple antibiotic resistance is becoming more common in sporadic ST7 GX strains and remains a threat to local public health. 

## 4. Materials and Methods

### 4.1. Bacterial Strains, Chromosomal DNA Sequencing and Bioinformatic Analysis

A total of 38 human clinical cases from eight regions of GX between 2007 and 2018 were reported. Among the 38 cases, 31 with epidemiological and clinical information were investigated. A total of 19 cases (61.3%) had been exposed to pigs or pork within one week of the appearance of the initial symptoms (Table 1). Most patients (67.7%, 21/31) had sepsis characterized by the acute onset of fever, chills, headaches, dizziness, malaise, abdominal pain, and diarrhea. One sepsis patient also had a coma. Nearly one-third of cases (29%, 9/31) had meningitis characterized by fever, headache, and neck stiffness. Five patients had a coma, two of which also had petechia and purpura. Only one case (a host of GX14) suffered STSLS with fever, hypotension, jaundice, pneumonia, petechia, purpura, coma, and multi-organ failure, which included liver, heart, and renal impairments. A total of 38 strains from human clinical cases from eight regions of GX between 2007 and 2018 were collected. Except for two strains, all had geographic information based on the place of each patient’s residence. Thirty cases were from four cities in the southeast of GX, including Yulin City (n = 18), Qinzhou City (n = 5), Beihai City (n = 4), and Guigang City (n = 3). The remaining six strains were from another four cities, with two each from Nanning City and Guilin City, and one each from Liuzhou City and Chongzuo City (Table 1). All strains were confirmed to belong to *S. suis* using 16S rRNA sequencing. The serotype was determined by an agglutination test using serum purchased from Statens Serum Institute (Copenhagen, Denmark). MLST and MCG typing were performed according to previously described methods [27,32]. The strains were sequenced by Illumina sequencing and sequences were assembled using SOAPdenovo (release1.04). Complete genomes of GX14, GX25 and GX28 were sequenced using PacBio and Illumina as representatives of sporadic ST7 strains, respectively. The information of sequences obtained in the study was provided in Table S1. Genes were predicted using Glimmer and gene orthologs were determined using OrthoMCL [8]. Sequence comparisons were performed using the blastN program within BLAST with an e-value cutoff e^−10^ and visualized using an in-house perl script.

### 4.2. Phylogenetic Analysis

In our previous study, 94 serotype 2 epidemic strains were clustered into six clades with clade 1 and clades 2 to 6 responsible for the outbreaks in 1998 and 2005, respectively [12]. Thirteen genomic sequencing read datasets (accession no. SRP064815) and one complete genome SC84 (accession no. FM252031) selected from each clade (2-3 genomes from each clade) as representatives of epidemic strains were included in the phylogenetic analysis. For comparison purposes, three complete genomes of the sporadic ST7 strains from diseased pigs from the two provinces with outbreaks were also included; these were the JS14 strain (accession no. CP002465, serotype 14, Jiangsu Province) [35], CS100322 strain (accession no. CP024050, serotype 2, Jiangsu province) and SC070731 strain (accession no. CP003922, serotype 2, Sichuan province) [36]. ST1 strain LOLA-SS002 (accession no. FIFC00000000.1, serotype 2, UK), available in GenBank, was used as outgroup to root the tree.

Single-nucleotide polymorphisms (SNPs) were detected using Bowtie2 and MUMmer v3.23 for sequencing reads and complete genomes, respectively, and the genome sequence of SC84 was used as a reference. SNPs were named by using our automatic pipeline described previously [8]. The high quality SNPs supported by more than 5 reads were then concatenated together according to the reference. The adjacent mutations within 5-bp were filtered, and the remained sequences were further checked by clonalFrameML to avoid recombination. The mutational SNP sites were then selected to construct a phylogenetic tree using the maximum likelihood method by FastTree v2.1.10. The Generalized Time-Reversible (GTR) was used to construct the Maximum Likelihood tree. The tree was presented using FigTree v1.4.0. 

The BEAST program v2.4.7 was used to estimate the divergence time of the lineages. Four clock models (strict clock, relaxed clock lognormal, relaxed clock exponential, and random local clock) and three population models (constant, exponential, and Bayesian skylines) were tested. The relaxed clock lognormal model in combination with the Bayesian skyline population model was used in accord with our previous study [12]. The chain length was set to 100,000,000 states with resampling every 10,000 states, and the first 1000 trees were ignored. All other parameters were set to default values.

### 4.3. Whole Genome Synteny Analysis 

MUMmer (version 3.22) and Lastz (version 1.02.00) were used to align reference genome SC84 and three complete genomes of GX14, GX25 and GX28. Genomic synteny was performed to identify insertions, deletions, translocations and inversions between genomes of epidemic and sporadic strains.

### 4.4. Detection of Antibiotic Resistance Determinants and Antimicrobial Susceptibility Profiles 

Antibiotic resistance genes were analyzed by searching the Comprehensive Antibiotic Resistance Database (CARD) and the Antibiotic Resistance Genes Database (ARDB). A resistance gene was only regarded as a homolog in tested strains if it showed at least 80% identity in protein sequence across 80% of the length of the protein [37]. Antimicrobial susceptibility testing was performed by assessing the minimum inhibitory concentration (MIC) for all isolates using MIC-test strip (Liofilchem, Roseto degli Abruzzi, Italy). MIC-test strips contained a gradient of concentrations of penicillin G (0.002–32 μg/mL), cefaclor (0.016–256 μg/mL), tetracycline (0.016–256 μg/mL), erythromycin (0.016–256 μg/mL), azithromycin (0.016–256 μg/mL), clindamycin (0.016–256 μg/mL), streptomycin (0.064–1024 μg/mL), kanamycin (0.016–256 μg/mL), spectinomycin (0.064–1024 μg/mL), gentamicin (0.016–256 μg/mL) and trimethoprim-sulfamethoxazole (0.002–32 μg/mL). *S. pneumoniae* ATCC 49619 was used for quality control. For penicillin G, cefaclor, tetracycline, azithromycin, erythromycin, clindamycin and trimethoprim–sulfamethoxazole, breakpoints were used as recommended in the 2016 Clinical and Laboratory Standard Institute (CLSI) Guidelines (M100-S26) for *Streptococcus spp. Viridans Group* and *Streptococcus pneumoniae*. No breakpoint values for streptomycin, kanamycin, gentamicin, and spectinomycin were available for *Streptococci*, and their breakpoints were taken from previous studies [38,39].

### 4.5. Nucleotide Sequence Accession Numbers

The sequences of the 38 *S. suis* strains sequenced in this study were deposited into GenBank under accession numbers ranging from SRR8523115 to SRR8523152.

## Figures and Tables

**Table 1:** The information of strains used in the study.

Lineage	Strain Name	Accession No.	City	Year	Symptom	PAI/GI	PEN G (BP: > 4 μg/mL)	CEF (BP: > 4 μg/mL)	TET (BP: > 8 μg/mL)	ERY (BP: > 1 μg/mL)	AZI (BP: >1 μg/mL)	CLI (BP: > 1 μg/mL)	STR (BP: > 250 μg/mL)	KAN (BP: > 250 μg/mL)	SPE (BP: > 256 μg/mL)	GEN (BP: > 250 μg/mL)	TRI (BP *: > 4 μg/mL)	Antibiotic Resistance Genes
Lineage 1	GX49	SRR8523142	Beihai	2015	Sepsis	ICESsuGX88	0.064	0.047	16	0.19	0.38	0.5	2	6	32	**6**	**1** *	*tet*(O)
	GX88	SRR8523131	Qinzhou	2018	Meningitis	ICESsuGX88	0.064	0.064	24	48	>256	>256	2	4	32	4	0.75 *	*tet*(O)
Lineage 2	GX47	SRR8523138	Yulin	2014	Sepsis	ICESsuGX88	0.064	0.064	24	>256	>256	>256	4	3	32	6	3 *	*erm*(B), *tet*(O)
Lineage 3	GX79	SRR8523125	Beihai	2017	Meningitis	/	0.047	0.047	24	>256	>256	>256	>1024	>256	16	12	0.5 *	*aph(3’)-IIIa*, *erm*(B), *tet*(O), *sat4*, *ant(6)-Ia*
Lineage 4a	GX14	SRR8523149	Qinzhou	2008	STSLS	72K	0.094	0.047	24	0.19	0.38	0.38	>1024	8	24	8	0.75 *	*ant(6)-Ia*, *tet*(M)
Lineage 5a	GX18	SRR8523148	Yulin	2011	Sepsis	ICESsuGX81	0.094	0.125	24	0.75	0.75	0.75	3	6	24	8	0.5 *	*tet*(O), *tet*(40)
	GX21	SRR8523152	Yulin	2012	Sepsis	CMGETZ080501	0.032	0.064	24	>256	>256	>256	512	>256	16	8	0.75 *	*aph(3’)-IIIa*, *erm*(B), *tet*(O), *tet*(40), *sat4*, *mefA*, *mel,ant(6)-Ia*
	GX22	SRR8523135	Chongzuo	2012	Sepsis	CMGEGX22	0.047	0.064	32	>256	>256	>256	>1024	>256	24	4	0.25 *	*aph(3’)-IIIa*, *erm*(B), *tet*(O), *tet*(40), *sat4*, *mefA*, *mel,ant(6)-Ia*
	GX98	SRR8523127	Guilin	2016	/	CMGEGX22	0.047	0.047	24	>256	>256	>256	>1024	>256	16	6	0.38 *	*aph(3’)-IIIa*, *erm*(B), *tet*(O), *tet*(40), *sat4*, *mefA*, *mel,ant(6)-Ia*
	GX9	SRR8523146	Guigang	2007	Meningitis	CMGETZ080501	1.5	2	16	>256	>256	>256	>1024	>256	24	3	>32 *	*aph(3’)-IIIa*, *erm*(B), *tet*(O), *tet*(40), *sat4*, *mefA*, *mel*
	GX25	SRR8523133	Yulin	2012	Sepsis	CMGETZ080501	0.047	0.064	16	>256	>256	>256	>1024	>256	24	6	3 *	*aph(3’)-IIIa*, *erm*(B), *tet*(O), *tet*(40), *sat4*, *mefA*, *mel,ant(6)-Ia*
Lineage 5b	GX24	SRR8523136	Yulin	2012	Meningitis	ICESsuGX81	0.064	0.094	16	>256	>256	>256	2	3	24	6	0.75 *	*erm*(B), *tet*(O), *tet*(40)
	GX37	SRR8523140	Guigang	2014	Sepsis	ICESsuGX81	0.047	0.094	24	0.75	0.75	0.5	6	4	48	8	0.5 *	*tet*(O), *tet*(40)
	GX89	SRR8523132	Guigang	2018	Meningitis	ICESsuGX81	0.064	0.064	24	0.5	0.5	0.75	2	3	24	2	0.38 *	*tet*(O), *tet*(40)
	GX91	SRR8523117	Liuzhou	2018	/	ICESsuGX81	0.047	0.064	16	>256	>256	>256	>1024	12	>1024	4	0.38 *	*spw, erm*(B), *tet*(O), *ant(6)-Ia, tet(40)*
	GX50	SRR8523121	Beihai	2015	Sepsis	ICESsuGX81	0.064	0.38	12	0.125	0.25	0.5	2	4	24	6	0.75 *	*tet*(O), *tet*(40)
	GX80	SRR8523124	Qinzhou	2017	Meningitis	ICESsuGX81	0.047	0.125	12	>256	>256	>256	2	4	24	4	0.75 *	*tet*(O), *tet*(40)
	GX48	SRR8523141	Yulin	2015	Sepsis	ICESsuGX81	0.064	0.064	16	0.5	0.75	0.75	6	8	24	8	1 *	*tet*(O), *tet*(40)
	GX27	SRR8523134	Yulin	2012	Sepsis	ICESsuGX81	0.064	0.125	16	0.38	0.38	0.75	2	2	48	4	0.5 *	*tet*(O), *tet*(40)
	GX70	SRR8523118	Beihai	2016	Sepsis	ICESsuGX81	0.047	0.19	16	0.38	0.5	0.75	2	2	32	4	0.19 *	*tet*(O), *tet*(40)
	GX85	SRR8523115	Qinzhou	2018	Meningitis	ICESsuGX81	0.064	0.064	16	0.75	0.75	0.75	2	3	48	4	1 *	*tet*(O), *tet*(40)
	GX81	SRR8523123	Nanning	2017	Sepsis	ICESsuGX81	0.094	0.094	12	48	>256	>256	8	12	24	6	2 *	*tet*(O), *tet*(40)
	GX87	SRR8523130	Qinzhou	2018	Sepsis	ICESsuGX81	0.047	0.094	24	>256	>256	>256	>1024	>256	32	>256	0.25 *	*AAC(6′)-Ie, APH(2″)-Ia, erm*(B), *tet*(O), *ant(6)-Ia, tet(40)*
	GX16	SRR8523150	Yulin	2010	Sepsis	ICESsuGX81	0.094	0.064	16	0.75	0.75	0.75	2	2	32	4	0.38 *	*tet*(O), *tet*(40)
	GX39	SRR8523137	Yulin	2014	/	ICESsuGX81	0.064	0.064	16	>256	>256	>256	>1024	>256	16	>256	>32 *	*dfrG, erm*(B), *tet*(O), *ant(6)-Ia, tet(40), AAC(6′)-Ie-APH(2″)-Ia*
	GX8	SRR8523145	Yulin	2007	Sepsis	ICESsuGX81	0.094	0.094	12	>256	>256	>256	3	8	24	6	0.25 *	*erm*(B), *tet*(O), *tet*(40)
	GX11	SRR8523143	Yulin	2008	Sepsis	ICESsuGX81	0.094	0.094	24	0.75	0.75	0.5	3	8	32	6	1 *	*tet*(O), *tet*(40)
	GX13	SRR8523144	Yulin	2008	Meningitis	ICESsuGX81	0.064	0.094	24	>256	>256	>256	4	6	32	4	0.25 *	*erm*(B), *tet*(O), *tet*(40)
	GX17	SRR8523147	Yulin	2010	Sepsis	ICESsuGX81	0.064	0.094	16	0.5	0.5	0.75	8	6	32	3	0.094 *	*tet*(O), *tet*(40)
	GX83	SRR8523122	Nanning	2017	Sepsis	ICESsuGX81	0.094	0.094	12	0.75	0.75	0.38	4	6	24	8	3 *	*tet*(O), *tet*(40)
	GX19	SRR8523151	Yulin	2011	Meningitis	ICESsuGX81	0.047	0.094	16	0.75	0.75	0.5	12	8	32	3	0.125 *	*tet*(O), *tet*(40)
	GX86	SRR8523129	Yulin	2018	Sepsis	ICESsuGX81	0.064	0.064	16	>256	>256	>256	>1024	8	>1024	3	0.25 *	*spw, erm*(B), *tet*(O), *ant(6)-Ia, tet(40)*
	GX28	SRR8523139	Yulin	2013	Sepsis	ICESsuGX81	0.047	0.064	16	>256	>256	>256	>1024	8	>1024	4	0.75 *	*spw*, *erm*(B), *tet*(O), *ant(6)-Ia, tet(40)*
	GX95	SRR8523128	/	2013	/	ICESsuGX81	0.047	0.064	24	>256	>256	>256	>1024	6	>1024	2	0.19 *	*spw, erm*(B), *tet*(O), *ant(6)-Ia, tet(40)*
	GX51	SRR8523120	Yulin	2015	Sepsis	ICESsuGX81	0.047	0.047	16	>256	>256	>256	>1024	2	>1024	8	0.75 *	*spw, erm*(B), *tet*(O), *ant(6)-Ia, tet(40)*
	GX64	SRR8523119	Yulin	2016	/	ICESsuGX81	0.094	0.064	16	>256	>256	>256	>1024	12	>1024	6	0.75 *	*spw, erm*(B), *tet*(O), *ant(6)-Ia, tet(40)*
	GX84	SRR8523116	Guilin	2017	/	ICESsuGX81	0.064	0.064	16	>256	>256	>256	>1024	8	>1024	3	0.5 *	*spw, erm*(B), *tet*(O), *ant(6)-Ia, tet(40)*
	GX97	SRR8523126	/	2018	/	ICESsuGX81	0.047	0.064	16	>256	>256	>256	>1024	4	>1024	1.5	0.38 *	*spw, erm*(B), *tet*(O), *ant(6)-Ia, tet(40)*

Abbreviations: PEN G = Penicillin G; CEF = Cefaclor; TET = Tetracycline; ERY = Erythromycin; AZI = Azithromycin; CLI = Clindamycin; STR = Streptomycin; KAN = Kanamycin; SPE = Spectinomycin; GEN = Gentamicin; TRI = Trimethoprim*-sulfamethoxazole (1/19) *: MIC value of Trimethoprim.

## Supplementary Materials

The following are available online at https://www.mdpi.com/2076-0817/8/4/187/s1, Table S1: The information of genomes sequenced in the study.
