# Supplementary material for: Genomic Epidemiology of Streptococcus suis Sequence Type 7 Sporadic Infections in the Guangxi Zhuang Autonomous Region of China
Source: Pathogens. 2019 Oct 12;8(4):187. doi: 10.3390/pathogens8040187 (PMC6963630; doi:10.3390/pathogens8040187)
Supplement: Supplementary file 1 [file pathogens-08-00187-s001.pdf]

Supplementary Table S1. The information of genomes sequenced in the study.

Strain	Number of reads	Number of base	Sequencing depth	Scaffold Length(bp)	Coverage (SC84 as reference) %	Scaffold GC(%)	Sum of SNPs (SC84 as reference)
GX8	10204190	1530628500	730.3	2066806	96.08	41.11	3978
GX9	11109292	1666393800	795.1	2111507	95.76	41.12	4918
GX11	9212064	1381809600	659.3	2066485	96.01	41.15	3837
GX13	10887088	1633063200	779.2	2101753	95.71	41.06	4797
GX14	9735670	1460350500	696.8	2069960	98.32	41.07	2473
GX16	8467688	1270153200	606.0	2062315	96.08	41.12	4876
GX17	9925380	1488807000	710.3	2101507	96.14	41.08	3901
GX18	10793910	1619086500	772.5	2064624	96.50	41.02	3793
GX19	10958510	1643776500	784.3	2053326	95.57	41.18	4221
GX21	6666668	1000000200	477.1	2119690	96.75	41.08	3131
GX22	8975214	1346282100	642.3	2122812	96.48	41.07	3744
GX24	8908540	1336281000	637.6	2120760	96.02	41.07	3811
GX25	9059542	1358931300	648.4	2120081	96.53	41.08	3738
GX27	11190488	1678573200	800.9	2056802	95.78	41.12	4698
GX28	9588596	1438289400	686.2	2068010	95.59	41.12	3802
GX37	9782898	1467434700	700.1	2063239	96.10	41.12	5661
GX39	8907642	1336146300	637.5	2122888	96.17	41.05	3719
GX47	8920832	1338124800	638.4	2035996	95.98	41.21	2680
GX48	9172632	1375894800	656.5	2062218	95.97	41.12	3742
GX49	10156500	1523475000	726.9	2032767	95.75	41.18	4657
GX50	8754526	1313178900	626.5	2062932	96.04	41.13	3800

GX51	6122078	918311700	438.1	2073177	95.67	41.1	4000
GX64	8632550	1294882500	617.8	2088039	95.66	41.08	4577
GX70	10824350	1623652500	774.7	2064799	96.10	41.12	4047
GX79	6666668	1000000200	477.1	2138184	97.02	40.98	2455
GX80	10299860	1544979000	737.1	2048336	95.40	41.19	5669
GX81	10614652	1592197800	759.7	2056293	95.78	41.14	4481
GX83	10384960	1557744000	743.2	2063873	96.08	41.11	4128
GX84	7293678	1094051700	522.0	2067497	95.62	41.11	4312
GX85	7861062	1179159300	562.6	2062335	96.03	41.12	4635
GX86	9222904	1383435600	660.1	2066540	95.58	41.12	5185
GX87	7998412	1199761800	572.4	2120096	96.19	41.05	4539
GX88	7590916	1138637400	543.3	2032333	95.70	41.19	5233
GX89	8294420	1244163000	593.6	2062182	96.08	41.12	4614
GX91	7358822	1103823300	526.7	2067684	95.64	41.11	4488
GX95	7940574	1191086100	568.3	2067280	95.64	41.11	4591
GX97	6917958	1037693700	495.1	2068703	95.72	41.11	4235
GX98	8038294	1205744100	575.3	2120606	96.38	41.07	4404
